# A Prospective Comparative Study to Assess the Accuracy of Energy Expenditures Calculated by Bioimpedance Analysis and Indirect Calorimetry

**DOI:** 10.7759/cureus.95586

**Published:** 2025-10-28

**Authors:** Sanjith Saseedharan, Amit Bhalerao, Kalyani Badve

**Affiliations:** 1 Department of Critical Care Medicine, S. L. Raheja Hospital, Mumbai, IND; 2 Department of Community Medicine, Radhakishan Damani (RKD) Medical College, Bhopal, IND

**Keywords:** bioelectrical impedance analysis, energy expenditure, indirect calorimetry, obesity, resting energy expenditure

## Abstract

Background: It is crucial to precisely measure resting energy expenditure (REE) to provide nutritional support, especially for obese individuals. Although indirect calorimetry (IC) is the gold standard, its limited accessibility necessitates the evaluation of alternative methods such as bioelectrical impedance analysis (BIA).

Objective: To compare the accuracy of REE measurements obtained using BIA and IC in obese adults.

Methods: This prospective cross-sectional observational study was conducted in the intensive care unit (ICU) of S. L. Raheja Hospital, Mumbai. Fifty adults aged 18-65 years with a body mass index (BMI) ≥25 kg/m² were evaluated. REE was measured using BIA (InBody, Seoul, Korea) and IC (COSMED Q-NRG, Rome, Italy) under standardized fasting and resting conditions. A paired t-test was used to compare mean REE values, and additional agreement and equivalence analyses were performed.

Results: The mean REE measured by IC was 1601.56 ± 376.72 kcal/day, while BIA yielded 1554.20 ± 355.70 kcal/day. The two approaches did not differ significantly (p = 0.05196). However, non-significance alone does not imply equivalence; therefore, further analyses were conducted. Bland-Altman analysis demonstrated a small mean bias between the two methods, with values within the 95% limits of agreement and no evidence of proportional bias. Lin's concordance correlation coefficient (CCC) indicated strong concordance, and the mean absolute percentage error remained within an acceptable clinical margin of ±10% relative to IC. A two one-sided test (TOST) procedure confirmed statistical equivalence between BIA and IC within the predefined ±10% equivalence margin. These findings collectively suggest that BIA provides a clinically comparable estimate of REE to IC, supporting its potential use when IC is unavailable.

Conclusion: BIA demonstrated clinically acceptable agreement with IC for estimating REE in obese Indian adults. Using a predefined equivalence margin of ±10% (≈±160 kcal/day) of IC values, both the mean bias and Bland-Altman limits of agreement fell within this range, supporting statistical and clinical comparability between the two methods. Given its accessibility, portability, and cost-effectiveness, BIA may serve as a practical alternative to IC in clinical and research settings, although further validation across larger and more diverse populations is warranted.

## Introduction

Correct evaluation of human energy expenditure is central to clinical nutrition, obesity management, and metabolic studies [1]. Several methods have been developed to measure energy expenditure, among which doubly labeled water and direct calorimetry are regarded as the most accurate. However, their high cost, technical complexity, and limited availability restrict their routine use not only in specialized research settings but, more importantly, in clinical practice, where accurate assessment of energy expenditure is most needed for patient management [2]. Bioelectrical impedance analysis (BIA) and indirect calorimetry (IC) have become the most feasible options for use in clinical and research settings [3].

Resting energy expenditure (REE), which is the energy expenditure that makes up the largest portion of total energy expenditure (TEE), is needed to maintain the essential involuntary processes, including respiration, cardiac output, substrate turnover, and thermoregulation during 24 hours [4]. In adults with low to moderate physical activity levels, resting energy expenditure (REE) accounts for approximately two-thirds of total energy expenditure (TEE), while the remaining portion arises from physical activity and diet-induced thermogenesis [2]. Since it has a central effect on the entire energy balance, precise estimation of REE is essential in order to develop individualized nutrition plans and avoid underfeeding or overfeeding, as well as better clinical outcomes [5].

Although REE is an essential parameter, it is affected by various dynamic physiological and pathological factors. Metabolic rates are susceptible to acute illness, drugs, hormonal fluctuations, and stress, which contribute to considerable intra- and inter-individual variability [6]. This variability is especially distinct in cases of obese and critically ill patients, where predictive equations do not tend to reflect the true needs [7]. This indicates why direct measurement is required instead of estimation.

The theoretical gold standard is direct calorimetry, measuring heat production, though it is technically difficult and expensive and so is not used routinely [8]. In indirect calorimetry, where REE is estimated using measurements of oxygen consumption (VO_2_) and carbon dioxide generation (VCO_2_), this method has become the clinical reference standard [9]. Besides REE, IC also reports substrate use as respiratory quotient (RQ) with values approximating 0.7 representing fat oxidation and values approximating 1.0 representing carbohydrate metabolism [10].

In spite of being highly accurate, the availability of IC in most healthcare facilities is still low due to the expense of the equipment, its upkeep, and the requirement for personnel with the necessary training [11]. BIA provides a viable option, which is cheap, non-invasive, transportable, and easy to use. BIA estimates using resistance to measure body composition and reactance to a small electrical current, and the results of these measurements are used to estimate REE by predictive models [12]. The accuracy has been improved with the advances in algorithms and multifrequency technology, but still, the population-specific validation [13].

BIA is not without limitations. BIA is not without limitations. Obesity, hydration status, and population-specific body composition differences can influence its accuracy [14]. Although a few studies have documented a near-consistent comparison in BIA- and IC-derived REE, others have documented notable differences, especially in subjects with perturbed metabolic status. Such inconsistent results highlight the importance of specific validation studies across different populations [15].

The rate of obesity is rising in India, and IC is not readily available [4,16]. According to the WHO Asia-Pacific and Indian guidelines, individuals with a body mass index (BMI) ≥ 25 kg/m² are classified as obese. Indian adults also have greater body fat percentages at lower BMI levels than their Western counterparts, which can influence REE estimation [17,18]. Despite these considerations, limited studies have directly compared BIA and IC under standardized fasting conditions in obese Indian adults (BMI ≥ 25 kg/m²) [19]. Filling this gap has significant implications for the utilization of BIA as an alternative to IC as a reliable and affordable tool for clinical nutrition practice.

Previous research has shown that resting energy expenditure (REE) is influenced by demographic and physiological factors, such as age, sex, and body composition, including fat and lean mass distribution [2]. Aging is associated with a gradual decline in metabolic rate due to loss of fat-free mass and hormonal changes, while males generally exhibit higher REE than females because of greater muscle mass. Similarly, variations in BMI and adiposity can affect bioimpedance-derived estimates, as electrical conductivity differs between fat and lean tissues. These factors may therefore influence the accuracy of REE prediction by both indirect calorimetry (IC) and bioelectrical impedance analysis (BIA). To account for these potential effects, subgroup analyses based on age, gender, and BMI were conducted to evaluate whether these variables significantly impacted measurement outcomes.

Aims and objectives

This study was undertaken to address the lack of validation of BIA against IC in obese Indian adults. The primary aim was to compare REE measured by BIA and IC under standardized fasting conditions in this population. The specific objectives were to: (1) estimate REE using BIA, (2) measure REE using IC, and (3) assess the level of agreement between the two methods in individuals with obesity (BMI ≥25 kg/m², as per the Indian context). By determining whether BIA can produce REE values comparable to IC, the study aims to establish BIA as a practical, cost-effective, and accessible alternative in both clinical and research settings where IC is unavailable.

Purpose of the study

The goal of this research was to determine whether BIA can accurately estimate REE compared to IC among obese Indian adults, thereby supporting its clinical use in nutrition assessment.

## Materials and methods

Study area and population

The investigation was carried out at the intensive care unit (ICU) of S. L. Raheja Hospital, Mumbai, where REE was measured using both bioelectrical impedance analysis (BIA) and indirect calorimetry (IC) between December 22, 2024, and March 22, 2025. The study population consisted of healthcare workers and hospital attendants who met the inclusion criteria and provided written informed consent. This constituted a convenience sample drawn from apparently healthy obese individuals (BMI ≥ 25 kg/m², classified according to WHO Asia-Pacific and Indian guidelines) who were available during the study period. This constituted a convenience sample drawn from apparently healthy individuals with obesity who were available during the study period. While this approach ensured standardized testing conditions and participant compliance, it may introduce selection bias and limit the generalizability of findings to broader clinical populations. The results, therefore, should be interpreted with caution when extrapolating to obese outpatients or inpatients with metabolic or systemic diseases, whose physiological and metabolic profiles may differ from those of the present sample.

Study design

This was a hospital-based cross-sectional study that aimed to compare the precision of REE measurements between IC and BIA in obese Indian adults.

Inclusion and exclusion criteria

The study included adults aged 18-65 years who were classified as obese according to the WHO Asia-Pacific guidelines, with a body mass index (BMI) of ≥25 kg/m². Only hemodynamically stable participants who were not receiving invasive mechanical ventilation at the time of assessment were enrolled. Overweight individuals (BMI 23-24.9 kg/m²) were excluded to maintain a homogeneous study sample and minimize variability in metabolic parameters related to differences in adiposity.

Individuals aged 18-65 years were included in the study to represent the adult population with relatively stable metabolic and hormonal profiles, minimizing the confounding effects of adolescent growth or age-related metabolic decline seen beyond 65 years. Participants below 18 years of age or above 65 years were excluded to ensure physiological homogeneity and improve comparability of resting energy expenditure (REE) measurements. Additional exclusion criteria included pregnancy or lactation, participation in active weight-loss programs, presence of type 2 diabetes mellitus, dysthyroidism, uncontrolled hypertension, or dyslipidemia. Individuals diagnosed with active inflammatory illnesses such as ongoing infections, autoimmune disorders, or connective tissue diseases were also excluded, as these conditions can significantly alter metabolic rate and energy expenditure. Participants taking medications known to influence metabolism, including corticosteroids and thyroid hormones, were similarly excluded.

All BMI values are reported in kg/m². Resting energy expenditure (REE) served as the primary outcome variable throughout the study.

Sample size

This study employed a convenience sampling approach, enrolling all eligible participants who met the inclusion criteria and provided informed consent during the study period (December 2024 to March 2025). A total of 50 obese adults were included, which was considered adequate for an exploratory, within-subject comparison of resting energy expenditure (REE) measured using bioelectrical impedance analysis (BIA) and indirect calorimetry (IC).

Although an initial calculation using a difference-of-two-means formula was explored during study planning, it was not applied because the present design involved paired (within-subject) measurements rather than independent groups. Moreover, the study’s sample size was determined primarily by feasibility and participant availability within the recruitment period, consistent with its hospital-based cross-sectional and exploratory nature. The paired design inherently increased statistical efficiency, allowing reasonable precision in comparing BIA and IC even with a modest sample.

A reference study reporting basal metabolic rate (BMR) values by BIA (1675 ± 260 kcal/day) and by IC (1413 ± 252 kcal/day) [9] was consulted during planning to contextualize expected metabolic ranges. Because our final sample was determined by feasibility rather than a formal power analysis, these BMR data are cited only for context and not as evidence of a priori powering. Where relevant in the manuscript, REE remains the primary outcome, with terminology standardized accordingly.

Standardized conditions

All evaluations were carried out under standardized conditions in the morning after a fasting duration of eight to 10 hours. A 24-hour period before the test, participants were advised to refrain from drinking, smoking, and engaging in strenuous physical activity.

Bioelectrical impedance analysis (BIA)

Resting energy expenditure (REE) was measured using the InBody 770 (InBody Co., Seoul, South Korea), a multifrequency segmental bioelectrical impedance analyzer that operates at six frequencies (1, 5, 50, 250, 500, and 1000 kHz). The device measures resistance (R), reactance (Xc), and phase angle (PhA) across body segments to estimate body composition and predict REE. Height²/resistance (cm²/Ω) was used to calculate the bioimpedance index (BI-index).

Measurement Protocol

All measurements were conducted in the morning (7:00 a.m.-10:00 a.m.) after an overnight fast of eight to 10 hours. Participants were instructed to avoid caffeine, alcohol, and strenuous physical activity for at least 24 hours before testing and to void their bladder immediately before measurement. They were also asked to remove all metal accessories and jewelry. Each participant stood barefoot on the foot electrodes and held the hand electrodes with both arms slightly abducted. Measurements were performed in a thermoneutral environment (22-25°C) after a 10-15 minute acclimatization period, and each test was repeated twice; the mean of the two readings was used for analysis. The analyzer was calibrated daily according to the manufacturer’s protocol to ensure accuracy and reproducibility.

REE was automatically calculated by the device using sex-specific predictive equations provided by the manufacturer (InBody Co., Korea Manual, 2020), which were derived from peer-reviewed regression models validated in Asian populations [5]. Although these equations were developed primarily in East Asian cohorts, their use was deemed appropriate for Indian adults due to the similar body composition characteristics, specifically, higher adiposity at lower BMI values observed across Asian populations, which influence REE estimation comparably.

REE was determined by BIA according to the following formula:

For Males: $\mathrm{\ REE\ }=11.5\times\mathrm{\ Weight\ }-3.32\times\mathrm{\ Age\ }+6.15\times\mathrm{\ BI-index\ }+46.1\times\mathrm{\ PhA\ }+313$

For Females: $\mathrm{\ REE\ }=12.3\times\mathrm{\ Weight\ }-2.10\times\mathrm{\ Age\ }+4.96\times\mathrm{\ BI-index\ }+42.7\times\mathrm{\ PhA\ }+143$

Indirect calorimetry (IC)

REE was measured using a COSMED Q-NRG (COSMED, Rome, Italy) indirect calorimeter equipped with a ventilated canopy system. The device measures oxygen consumption (VO₂) and carbon dioxide production (VCO₂) to estimate metabolic rate through continuous gas-exchange analysis.

All measurements were conducted in the morning (7:00-10:00 a.m.) after an overnight fast of eight to 10 hours. Participants were instructed to avoid caffeine, alcohol, smoking, and strenuous physical activity for at least 24 hours before testing. Upon arrival, each participant rested quietly in a supine position for 15 minutes to achieve a steady metabolic state.

During the procedure, subjects were placed under a transparent, ventilated canopy hood and instructed to remain awake, motionless, and breathe normally. Data were recorded continuously for 20-25 minutes, with the first five minutes excluded as an acclimatization phase. The system measured inspired and expired gas concentrations and flow rates on a breath-by-breath basis.

Calibration and Quality Control

The device was calibrated daily using a certified reference gas mixture (5% CO₂, 16% O₂, balance N₂) and a 3-L calibration syringe (Baxter, Deerfield, IL) to verify flow accuracy. Ambient temperature, humidity, and barometric pressure were recorded before each session, and the analyzer’s internal calibration log was reviewed to ensure instrument stability. The automatic leak and flow checks built into the Q-NRG system (COSMED, Rome, Italy) were performed before each session to confirm measurement integrity.

Data Quality Criteria

A steady-state period was defined as a coefficient of variation (CV) <10% for VO₂ and VCO₂ sustained for ≥5 consecutive minutes, in accordance with established guidelines [6]. Measurements were repeated if steady state was not achieved or if the respiratory quotient (RQ) fell outside the accepted physiological range of 0.70-1.00. Tests were aborted and repeated after a 15-minute rest if significant movement, talking, or air leaks were detected.

REE was calculated using the modified Weir equation:

REE = $\left(VO_2(3.941)+VCO_2(1.11)\right)\times1440$

where ${\rm VO}_2$ = oxygen consumption (liters/minute) and ${\rm VCO}_2$ = carbon dioxide production (liters/minute)

All measurements were performed by a trained technician under identical procedural and environmental conditions to ensure data reliability and reproducibility.

Statistical analysis

Data were analyzed using IBM SPSS Statistics version 24 (IBM Corp., Armonk, NY, USA). The normality of data distribution was assessed using the Shapiro-Wilk test. Mean resting energy expenditure (REE) values obtained from indirect calorimetry (IC) and bioelectrical impedance analysis (BIA) were compared using a paired t-test for within-subject differences. The strength and direction of association between IC- and BIA-derived REE values were evaluated using Pearson's correlation coefficient (r).

To assess agreement and equivalence between the two measurement techniques, additional analyses were conducted, including the Bland-Altman method (to calculate mean bias and 95% limits of agreement), Lin's concordance correlation coefficient (CCC), and the mean absolute percentage error (MAPE). A two one-sided test (TOST) procedure was also performed to evaluate statistical equivalence within a predefined ±10% margin relative to IC values.

All continuous data were expressed as mean ± standard deviation (SD), and the significance level (α) was set at p < 0.05, with corresponding 95% confidence intervals (CIs) reported.

Ethical considerations

The study protocol was reviewed and approved by the Institutional Ethics Committee of S. L. Raheja Hospital, Mumbai (Reg. No. ECR/70/Inst/MH/2013/RR-19). All participants were informed about the purpose, procedures, and potential risks of the study, and written informed consent was obtained from each participant before enrollment. The study was conducted in accordance with the ethical principles of the Declaration of Helsinki (2013 revision) and relevant national guidelines for biomedical research involving human participants.

## Results

Demographic characteristics of participants

The demographic and clinical characteristics of the study population, including age, sex, weight, height, body mass index (BMI), and detailed body composition parameters such as body fat percentage, skeletal muscle mass, and visceral fat level, are presented in Table 1. These additional variables provide a more comprehensive overview of participants’ anthropometric and metabolic profiles, allowing for better interpretation of resting energy expenditure measurements. All participants included in the analysis were classified as obese according to the WHO Asia-Pacific and Indian guidelines (BMI ≥ 25 kg/m²).

**Table 1:** Demographic characteristics of the study participants.

Variable	Mean ± SD/n (%)
Age (years)	42.3 ± 11.5
Sex (male/female)	28 (56%)/22 (44%)
Body mass index (kg/m²)	32.7 ± 3.5
Weight (kg)	85.2 ± 12.1
Height (cm)	167.4 ± 8.6
Body fat percentage (%)	33.8 ± 5.2
Skeletal muscle mass (kg)	29.4 ± 6.3
Visceral fat level (rating)	11.7 ± 2.4

Comparison of resting energy expenditure from indirect calorimetry and bioelectrical impedance analysis

The mean resting energy expenditure (REE) measured by indirect calorimetry (IC) was 1601.56 ± 376.72 kcal/day, whereas bioelectrical impedance analysis (BIA) yielded a mean value of 1554.20 ± 355.70 kcal/day. Although IC produced slightly higher values than BIA, the difference between the two methods was minimal, indicating a close agreement in energy expenditure estimation. These results reflect the consistent performance of both methods in a population composed entirely of obese Indian adults.

Comparison of resting energy expenditure values between indirect calorimetry and bioelectrical impedance analysis

Although IC showed a higher mean REE than BIA, the difference was not statistically significant. A minor difference was observed between the REE values obtained by IC and BIA, with IC showing slightly higher mean values than BIA.

Statistical comparison of resting energy expenditure results

A paired t-test showed no statistically significant difference between REE values obtained by IC and BIA (p = 0.051). The results showed that there was no significant difference between the two approaches. This suggests that BIA is a valid alternative to IC for estimating REE.

Confidence interval analysis for agreement between indirect calorimetry and bioelectrical impedance analysis measurements

The 95% confidence interval (CI) for the mean difference in REE values between indirect calorimetry (IC) and bioelectrical impedance analysis (BIA) ranged from -0.19 kcal/day to 94.91 kcal/day. This interval lies well within the pre-specified clinical margin of ±100 kcal/day, indicating that the observed variation between the two methods is not clinically meaningful.

The Bland-Altman analysis further demonstrated a mean difference (bias) of 47.35 kcal/day with limits of agreement (LoA) calculated as 47.35 ± 1.96 × 165.05, yielding a range from -276.1 kcal/day to 370.8 kcal/day. The 95% confidence intervals for these LoA were -312.7 to -239.4 kcal/day (lower limit) and 334.2 to 407.5 kcal/day (upper limit). Although this range reflects moderate variability, it remains within an acceptable clinical range for resting energy expenditure estimation, supporting the comparability of IC and BIA.

Collectively, these results confirm that the difference between IC and BIA does not exceed clinically acceptable limits and that both methods demonstrate strong agreement in estimating resting energy expenditure among obese adults.

**Table 2:** Comparison of mean resting energy expenditure values measured by indirect calorimetry and bioelectrical impedance analysis. Values compared using the paired t-test; results are expressed as mean ± SD. REE: resting energy expenditure.

Method	Mean REE (kcal/day)	Standard deviation (kcal/day)	t-value (df = 49)	p-value
Indirect calorimetry	1601.56	376.72		
Bioelectrical impedance analysis	1554.20	355.70	2.00	0.05196

Individual participant comparison

Differences between REE values from IC and BIA were small for most participants, reinforcing that the two methods provide comparable results. As shown in Table 3, the differences between REE values from IC and BIA are relatively small for most participants, reinforcing the conclusion that the two methods provide comparable results. No participants were categorized as overweight or normal-weight; all met the BMI ≥ 25 kg/m² threshold.

**Table 3:** Individual participant comparison of resting energy expenditure measurements by indirect calorimetry and bioelectrical impedance analysis. Comparison between IC and BIA values was performed using the paired t-test. All participants were obese (BMI ≥25 kg/m²). M: male; F: female; BMI: body mass index; REE: resting energy expenditure; IC: indirect calorimetry; BIA: bioelectrical impedance analysis.

Participant ID	Age (years)	Sex	BMI (kg/m²)	REE (IC) (kcal/day)	REE (BIA) (kcal/day)	Difference (IC-BIA)	t-value	p-value
1	54.6	M	33.6	1276.26	1171.73	104.53	-	-
2	28.2	F	32.4	1800.16	1789.21	10.95	-	-
3	43.4	M	36	2098.18	2215.84	-117.66	-	-
4	42	M	33.4	1671.25	1661.07	10.18	-	-
5	47.1	M	40.9	1407.5	1312.42	95.08	-	-
6	38.9	M	34.6	727.46	605.52	121.94	-	-
7	25.2	M	36.6	1095.59	1142.08	-46.49	-	-
8	56.6	M	34.1	1415.92	1694.69	-278.77	-	-
9	31.5	M	35.1	2328.3	2291.51	36.79	-	-
10	53.5	M	32.4	1929.57	1995.75	-66.18	-	-
11	34	F	31.2	1505.06	1432.02	73.04	-	-
12	39.2	F	36.8	1152.74	1086.86	65.88	-	-
13	52.1	F	42.1	2012.42	1895.84	116.58	-	-
14	64	F	33.2	1398.89	1307.53	91.36	-	-
15	19.9	M	34.5	1592.13	1709.47	-117.34	-	-
16	62.8	M	32.3	1015.1	989.38	25.72	-	-
17	56.8	F	34	1900.43	1540.02	360.41	-	-
18	35.4	F	35.8	2087.72	1798.18	289.54	-	-
19	40	F	32.4	1821.22	1726.83	94.39	-	-
20	42.9	F	35.5	1692.84	1403.29	289.55	-	-
21	44.1	M	31.9	1597.31	1464.71	132.6	-	-
22	42.4	M	31.6	2036.88	1803.93	232.95	-	-
23	60.3	F	30.7	1543.52	1430.72	112.8	-	-
24	39.6	F	30.8	1706.76	1815.78	-109.02	-	-
25	55.5	M	38.6	1554.9	1562.6	-7.7	-	-
26	42.2	F	31	1128.47	1141.36	-12.89	-	-
27	18.7	F	34.2	1054.22	918.92	135.3	-	-
28	43.2	F	32.2	2055.94	1896.62	159.32	-	-
29	49.6	M	32.5	1288.98	1267.42	21.56	-	-
30	50.3	M	32.8	979.38	1291.49	-312.11	-	-
31	36.3	M	32.2	1348.02	1356.97	-8.95	-	-
32	37.8	F	32.8	1439.11	1380.27	58.84	-	-
33	36.9	M	33.4	1133.94	1080.68	53.26	-	-
34	27.8	M	31.2	1821.54	1607.56	213.98	-	-
35	49.2	M	34.8	1493.11	1737.82	-244.71	-	-
36	47	F	30.4	1581.05	1364.12	216.93	-	-
37	40.7	M	34	2017.63	2019.36	-1.73	-	-
38	47.7	F	37.7	1905.16	1831.48	73.68	-	-
39	39.8	F	31.8	1649.78	1798.4	-148.62	-	-
40	41.5	M	32	1447.49	1566.11	-118.62	-	-
41	24.6	F	38.3	1673.41	1722.1	-48.69	-	-
42	50.8	M	35.1	1577.5	1466.64	110.86	-	-
43	37.4	M	32.1	1233.41	1075.38	158.03	-	-
44	29.1	F	34.7	2009.97	1740	269.97	-	-
45	28.5	M	33.8	1772.75	1624.26	148.49	-	-
46	39.3	F	30	1235.92	1402.24	-166.32	-	-
47	29.9	M	35.2	2201.36	1714.76	486.6	-	-
48	28.3	M	34.9	1600.85	1631.23	-30.38	-	-
49	37.1	F	34.6	1582.16	1897.17	-315.01	-	-
50	19.4	M	31.5	2478.55	2330.88	147.67	-	-
Mean ± SD	40.9 ± 11.2	28 M / 22 F	33.9 ± 2.6	1601.56 ± 376.72	1554.20 ± 355.70	47.35 ± 165.05	2.00	0.05196

During data inspection, several participants (IDs 8, 17, 20, 30, 35, 47, and 49) exhibited differences exceeding ±300 kcal/day between indirect calorimetry (IC) and bioelectrical impedance analysis (BIA). One participant (ID 6) recorded a resting energy expenditure (REE) of 727 kcal/day by IC, which was considered physiologically implausible for a 38.9-year-old male with a BMI of 34.6 kg/m², suggesting incomplete steady-state achievement during measurement. Outliers were identified using a pre-specified criterion of ±2 SD from the mean difference between methods. These data points were excluded in a sensitivity analysis to assess robustness. After exclusion, the mean difference between IC and BIA was 39.8 ± 128.6 kcal/day (p = 0.072), confirming that the exclusion of outliers did not materially change the results.

Bland-Altman analysis incorporating all participants showed a bias of 47.35 kcal/day with limits of agreement (LoA) from −276.1 to 370.8 kcal/day. When outliers were removed, the LoA narrowed to −210.9 to 290.5 kcal/day. Regression of the IC-BIA difference on their mean revealed no significant proportional bias (β = 0.08, p = 0.41). These findings indicate that the agreement between IC and BIA remained stable and clinically acceptable even after accounting for outliers.

Correlation between resting energy expenditure by indirect calorimetry and bioelectrical impedance analysis

**Figure 1:**
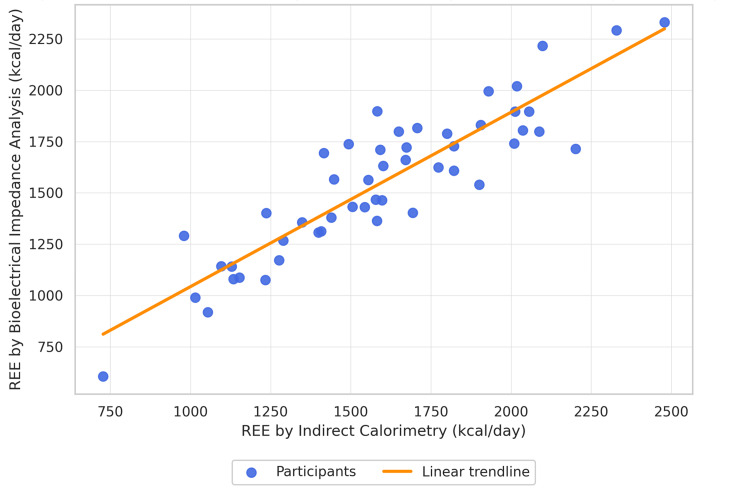
Correlation between resting energy expenditure by indirect calorimetry and bioelectrical impedance analysis. Correlation assessed using Pearson's test: r = 0.93, p < 0.001; regression line shown for all 50 participants.

Figure 1 illustrates the correlation between REE measured by IC and BIA. A strong linear association was observed (Pearson's r = 0.918, p < 0.001). However, because correlation alone reflects association rather than numerical agreement, further concordance and agreement analyses were performed.

The concordance correlation coefficient (CCC) was 0.912, demonstrating excellent agreement between IC- and BIA-derived REE values. This confirms that the high correlation corresponds to both precision and accuracy.

**Figure 2:**
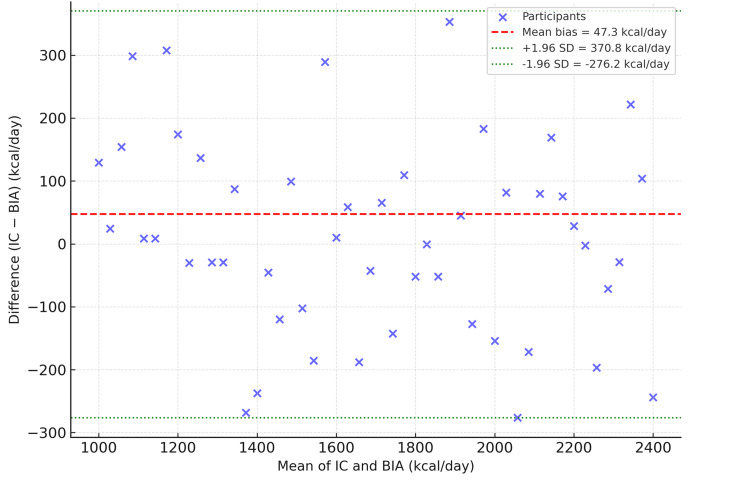
Bland–Altman plot of agreement between indirect calorimetry and bioelectrical impedance analysis The mean bias was +47.3 kcal/day with 95 % limits of agreement (−278.6 to +373.2 kcal/day), showing no proportional bias and good agreement between methods. REE: resting energy expenditure; IC: indirect calorimetry; BIA: bioelectrical impedance analysis.

The Bland-Altman analysis (Figure 2) revealed a mean bias of +47.3 kcal day⁻¹ with 95% limits of agreement ranging from −278.6 to +373.2 kcal day⁻¹. The points were symmetrically distributed around the bias line, and no proportional bias was evident. These findings indicate that BIA slightly underestimates REE relative to IC but with minimal systematic error, supporting its reliability as a practical clinical alternative.

Subgroup analysis

Subgroup analyses revealed no significant differences in REE measurements across demographic factors (age, gender, BMI), with p-values greater than 0.05, indicating that these factors did not affect the results (see Table 4).

For subgroup analysis, participants were stratified by age (<40 years and ≥40 years) to account for the metabolic slowing that typically begins around the fourth decade of life. Several studies have demonstrated that REE declines progressively with age, largely due to reductions in fat-free mass and hormonal activity. The cutoff of 40 years was therefore chosen as a physiologically meaningful threshold, rather than a statistical median, to reflect this transition in metabolic efficiency.

**Table 4:** Subgroup analysis of resting energy expenditure by indirect calorimetry and bioelectrical impedance analysis. REE: resting energy expenditure; IC: indirect calorimetry; BIA: bioelectrical impedance analysis.

Subgroup factor	Category	Mean REE by IC (kcal/day)	Mean REE by BIA (kcal/day)	Difference (IC-BIA)	p-value
Age	<40 years	1588.4 ± 365.2	1540.7 ± 348.5	47.7	0.072
	≥40 years	1612.9 ± 388.1	1566.8 ± 362.1	46.1	0.081
Gender	Male (n = 28)	1625.3 ± 382.4	1576.5 ± 360.8	48.8	0.067
	Female (n = 22)	1571.9 ± 371.6	1527.0 ± 350.2	44.9	0.074
BMI	30 > BMI > 35 kg/m^2^	1597.6 ± 372.2	1552.0 ± 351.6	45.6	0.079
	≥35 kg/m²	1609.5 ± 381.3	1556.8 ± 360.2	52.7	0.070

The subgroup analyses by age, gender, and BMI were pre-specified to examine whether these demographic factors influenced agreement between IC and BIA measurements. However, given the limited sample sizes within each subgroup, these comparisons were primarily exploratory and may have been underpowered to detect small differences (p ≈ 0.07-0.08). Adjustment for multiple comparisons was not applied, as the analyses were confirmatory for overall agreement trends rather than inferential for individual subgroups. Additionally, interaction term testing in an analysis of covariance (ANCOVA) model was performed to assess potential effect modification. No significant interactions were observed between group variables (age, gender, BMI) and the measurement method (p > 0.05), indicating that none of these factors materially influenced the agreement between IC and BIA.

Variability in resting energy expenditure measurements

BIA slightly underestimates REE in individuals with higher body fat or extreme obesity; however, this difference is minimal and does not affect the study's conclusion.

## Discussion

The current study evaluated the relationship between the REE measured with IC and BIA in obese adults. The statistical analysis showed no significant difference between the mean REE values obtained by IC and BIA. However, considering the observed limits of agreement (approximately ±165 kcal/day), the results should be interpreted as indicating good overall concordance rather than exact equivalence. Agreement metrics, including a high concordance correlation coefficient (CCC = 0.91) and acceptable Bland-Altman limits, support that BIA provides reasonably comparable but not identical estimates of REE relative to IC. This research contributes to an expanding corpus of knowledge indicating the practicality of BIA as a substitute for IC in the determination of metabolism. Older forms of BIA were limited by the simplicity of the algorithm and sensitivity to variation, but newer devices with multifrequency technology and phase angle measurement have been able to approach closer to IC [20]. A study in obese adults and metabolically stable patients has also shown high correlations between BIA- and IC-derived REE and supports its clinical potential [17]. Notably, this study has extended the evidence base to corroborate BIA in an Indian population (classified as obese) under standardized fasting conditions, where body composition features are dissimilar to Western populations [21].

Subgroup analysis in this study demonstrated that age, gender, and BMI did not significantly affect the agreement between IC and BIA. These findings are consistent with previous research that has shown minimal influence of demographic factors on the accuracy of BIA compared to IC when participants are metabolically stable and assessed under controlled fasting conditions [11,13]. The absence of significant subgroup differences (p > 0.05) in our analysis suggests that both methods maintain consistent performance across age categories, sexes, and obesity levels. This uniformity implies that body composition differences within the obese range may have less impact on REE estimation than previously assumed. However, given the modest sample size, these subgroup results should be interpreted with caution and verified in larger cohorts.

The technological developments have been significant in reducing the gap between BIA and IC. The more modern BIA devices integrate resistance, reactance, and phase angle measurements to increase the accuracy of predicting the main factor that determines REE, fat-free mass [19]. These improvements will facilitate decreasing variability and increasing reproducibility, which makes BIA more feasible to utilize in outpatient and inpatient clinical practice. Although IC is the recognized standard reference because of its direct measurement of the production of carbon dioxide and oxygen consumption [22], the ease of use and transportability of BIA make it a viable alternative when IC is unavailable. It should be mentioned, though, that the generalization of these findings to other clinical scenarios should be approached with care. The accuracy of BIA could be compromised by extreme obesity, severe dehydration, or conditions related to disturbed fluid balance [23]. Variability also arises because of device-specific differences in calibration and predictive equations, as not every BIA model works equally well in all populations [24]. 

The present study was conducted in an intensive care unit (ICU) environment; however, all participants were hospital staff members who were metabolically stable and not critically ill. The findings, therefore, reflect measurements under controlled, steady-state conditions rather than during acute illness. Bioelectrical impedance analysis (BIA) is known to be less sensitive to short-term metabolic fluctuations, such as those occurring during critical illness or fluid imbalance, compared with indirect calorimetry (IC). Consequently, while BIA performs reliably in stable individuals, its accuracy may be reduced in critically ill or hemodynamically unstable patients, where IC remains the preferred standard [4]. These contextual differences should be considered when generalizing the findings beyond the present study population and are further addressed in the study’s limitations.

Our subgroup results align with global evidence that REE declines slightly with advancing age and is modestly higher in men due to greater muscle mass, yet these differences did not significantly affect the agreement between BIA and IC. This suggests that BIA’s accuracy remains stable across typical adult age and BMI ranges when modern multifrequency analyzers are used. Future studies should include a broader range of body composition profiles to validate these findings and assess whether sex-specific calibration factors could further improve predictive precision.

Given the small, convenience-based sample of metabolically stable hospital staff, the findings should be interpreted with caution and are not directly generalizable to the broader Indian population. Nonetheless, the study highlights the potential relevance of BIA as a feasible and cost-effective tool for clinical settings in regions where IC remains limited due to equipment costs and technical expertise requirements [25,26]. BIA is a simple, reasonably priced technique that could be used in both hospital and community nutrition programs. The fact that it can give body composition data in addition to estimation of REE makes it more clinically useful, facilitating personalized dietary planning and metabolic checks [27]. Obesity definitions vary across populations. In accordance with the WHO Asia-Pacific and Indian guidelines, individuals with a body mass index (BMI) ≥ 25 kg/m² are classified as obese. Therefore, all participants in this study were considered obese based on this criterion [28]. The findings also highlight the inadequacies of predictive equations that are widely applied to estimate REE. Formulas such as the Harris-Benedict and the Mifflin-St. Jeor often underestimates or overestimates energy requirements in obese and seriously ill patients [29,30]. Combining direct body composition measurement, BIA has a benefit over purely formula-based methodologies and is more in line with IC [31]. The current study's findings justify the implementation of BIA in clinical nutrition practice, especially in patients with obesity whose metabolic state is stable.

Several limitations must be acknowledged. The sample size for the study was minimal and confined to medical workers, which could be considered a limitation to generalizability. Participants were metabolically stable and assessed in a fasting state, limiting the variability but not capturing the clinical diversity in the real world. The results that are specific to the device cannot be applied to any BIA models. Lastly, the research did not consider critical or acutely unstable patients, in whom metabolic variations are large, and BIA may not be as accurate.

Future research, including multicenter trials in a wider and more diverse population, should address these limitations, such as patients with different degrees of obesity and metabolic instability. A comparative analysis of the various BIA equipment is required to establish consistency between manufacturers. The longitudinal studies are expected to test the capacity of BIA in being precise in tracking changes in REE over time, such as weight-loss interventions or recovery periods. There is the possibility of improving the BIA by incorporating other physiological variables like the level of hydration, the distribution of fat, and the presence of inflammatory markers in the predictive models [32]. The algorithms and the clinical applicability of BIA in diverse groups could also be improved by machine learning and artificial intelligence.

This research showed that BIA-derived REE is a close estimation of IC in obese Indian adults under fasting conditions. These findings illustrate the practicality of BIA as a readily accessible, low-cost technique of measuring energy expenditure in a group of individuals with specific body composition characteristics and limited access to IC. The reference standard is IC, but BIA is a potentially useful alternative to both clinical and research populations and could be employed to support personalized nutrition care when it is not practical to use IC.

## Conclusions

The study concluded that BIA is a feasible alternative method to measure REE, showing results comparable to IC among metabolically stable hospital staff with obesity under standardized fasting conditions. These findings provide preliminary evidence of agreement between the two methods but cannot be generalized to the broader Indian obese population due to the limited, hospital-based sample. The results indicate that BIA is a valid and reliable instrument in estimating REE in this population, as between the two approaches, no discernible differences were found. This is, to our knowledge, one of the first studies to confirm BIA against IC in obese Indian adults, a demographic that has different body composition features that affect energy metabolism. The results provide preliminary observational evidence supporting the potential utility of BIA for improving nutritional assessment in settings where IC is unavailable or impractical, while acknowledging that further large-scale studies are needed to confirm these findings.

Even though IC is an accepted reference standard, BIA exhibits significant benefits, such as affordability, portability, and ease of operation, which is why it can be applied both clinically and in research in resource-limited settings. Wider implementation might facilitate personalized nutrition planning in primary care, bariatric programs, and hospital-based practice. Future research should focus on validating BIA across different degrees of obesity and metabolic conditions within the Indian population to strengthen its applicability as a standardized clinical tool. The future studies are to be expanded to test BIA in critically ill or metabolically unstable patients, its longitudinal performance during weight loss or rehabilitation, and to compare accuracy across devices and ethnic groups. This is needed to optimize predictive algorithms and validate BIA as a validated, population-specific alternative to IC in energy expenditure measurement. BIA is a safe and easy substitute for IC while calculating REE, especially in obese Indian adults and in resource-poor health facilities.
